# Artificial Intelligence-Augmented Standardized Patient Models for AETCOM (Attitude, Ethics, and Communication) Competency Evaluation: A Pilot Study

**DOI:** 10.7759/cureus.112683

**Published:** 2026-07-14

**Authors:** Nayyar Iqbal, Magi Murugan, Sunil Subramanyam, Sandesh Venu, Manikandan Mani, Renu G'Boy Varghese, Ramachandran Thiruvengadam

**Affiliations:** 1 General Medicine, Pondicherry Institute of Medical Sciences, Puducherry, IND; 2 Anatomy, Pondicherry Institute of Medical Sciences, Puducherry, IND; 3 Forensic Medicine and Toxicology, Pondicherry Institute of Medical Sciences, Puducherry, IND; 4 Psychiatry and Behavioral Sciences, Pondicherry Institute of Medical Sciences, PUDUCHERRY, IND; 5 Community Medicine, Pondicherry Institute of Medical Sciences, Puducherry, IND; 6 Pathology, Pondicherry Institute of Medical Sciences, Puducherry, IND; 7 Biochemistry, Translational Health Science and Technology Institute, Faridabad, IND

**Keywords:** ai-based assessment, ai chatbot, attitude ethics and commuication, attitude ethics and communication (aetcom), communication skill, informed consent, personalized learning, soft skill assessment, virtual standardized patient

## Abstract

Introduction: Communication skills are a core competency in the AETCOM (Attitude, Ethics, and Communication) module of the Competency-Based Medical Education curriculum. They are essential for building trust, taking accurate history, explaining interventions, obtaining informed consent, showing empathy, and managing challenging situations. Traditional assessments, such as Objective Structured Clinical Examinations (OSCEs), standardized patients, and mini clinical evaluation exercise (mini-CEX), are reliable but resource-intensive, are limited in frequency, and often provide delayed feedback.

Method: The study was conducted at Pondicherry Institute of Medical Sciences, Puducherry, India, after obtaining approval from the Institutional Research and Ethics Committee. Following expert discussion, informed consent was selected as the AETCOM competency to assess. To achieve the study objectives, two AI chatbots were developed: a virtual standardized-patient chatbot simulating a spinal injury scenario and an AI-based evaluation chatbot incorporating a 19-item content-validated scoring rubric. Thirty-three interns interacted with the virtual patient, and anonymized transcripts were independently evaluated by the AI system and five subject experts. AI reliability was assessed using intraclass correlation coefficients (ICCs), and agreement between AI and expert scores was evaluated using Bland-Altman analysis.

Result: Expert ratings showed moderate reliability individually (ICC (2, 1) = 0.536) but improved significantly when averaged (ICC (2, 5) = 0.852), highlighting the benefit of consensus scoring. AI scoring demonstrated excellent internal reliability (ICC (2, 1) = 0.904; ICC (2, 5) = 0.979) across repeated assessments. Bland-Altman analysis revealed a small negative bias (-0.529) with acceptable limits of agreement (-1.998 to 0.940), indicating close alignment with expert consensus.

Conclusion: The findings show that AI is highly reproducible, consistent, and standardized. AI virtual patients provide immediate feedback, support repeated practice, and reduce variability in assessment. Combined with expert assessment, AI-based systems may offer a scalable, standardized, and learner-centred approach to communication-skills assessment that warrants further evaluation in larger multicentre studies. This approach has the potential to enhance communication-skills training and assessment by providing standardized, reproducible, and timely feedback. Further studies are required to evaluate its impact on learner performance and clinical practice.

## Introduction

Communication skills form the core competency of the AETCOM module (Attitude, Ethics, and Communication) in the Competency-Based Medical Education (CBME) curriculum introduced by the National Medical Commission (NMC) of India in 2019. CBME emphasizes the development of an Indian medical graduate who is not only clinically competent but also empathetic, ethical, and an effective communicator with patients and their families. This curriculum identifies communication skills as a trainable and assessable competency, so they are integrated longitudinally across all phases of the medical curriculum [1].

Effective communication is essential for building trust with the patient, eliciting accurate history, ensuring informed consent, and engaging in shared decision-making, showing empathy and involving compliance with treatment. Good communication also enables students to handle sensitive situations like bad news, deal with angry patients, resolve conflicts, and work effectively with healthcare teams. Communication skills transform students into compassionate, patient-centred doctors who provide holistic care [2].

Traditionally, communication skills in medical education are assessed using objective structured clinical examinations (OSCEs), standardized patient (SP) encounters, and workplace-based assessments. OSCEs are widely used as a reliable and structured method to assess observable communication skills, such as empathy, clarity in the explanation of patient doubts, expression of the necessary information, and engagement of the patient in making decisions using checklists and rating scales [3]. Simulation-based assessment enhances realism and allows evaluation of the doctor-patient interaction, including informed consent and counselling skills [4]. In clinical settings, workplace-based assessments, such as the mini clinical evaluation exercise (mini-CEX), assess communication skills in various scenarios, like with patients, relatives, peers, juniors, and seniors, with emphasis on feedback. It has a higher fidelity as it permits evaluation based on a broader set of clinical settings and patient problems and is administered on site [5].

In spite of its widespread use, these approaches have their own limitations. OSCEs and SP are resource-intensive, requiring trained actors, many faculty, and logistical coordination, which limits scalability and frequency of assessment. Inter-rater variability and subjective interpretation of the checklists can compromise reliability, especially for empathy and professionalism. Work-based assessment often suffers from disadvantages, like inconsistent observation, limited documentation, and time constraints, reducing their educational impact. In addition, these methods provide only episodic and delayed feedback, making it difficult to support continuous programmatic assessment of communication competencies [6].

Recent advancements in artificial intelligence (AI), including large language models (LLMs) and virtual simulation environments, present a potential to transfer and augment traditional simulated patient assessments by enabling dynamic, repeatable, and contextually rich virtual patient interactions. AI-simulated patients can interact with students anytime in a safe and nonjudgmental environment. They can present realistic clinical scenarios such as history taking, informed consent, counselling, or breaking bad news, which are the key components of AETCOM. Because every learner faces the same scenario, the assessment is standardized and fair.

During the interactions, the AI system can automatically analyze how the students have communicated, such as clarity of explanations, use of respectful language, and response to the patient’s emotions. Based on this analysis, the AI can generate objective scores and immediate feedback, helping learners to understand what they did well and areas of improvement. An AI-simulated patient also allows repeated practice, which supports continuous learning and improvement. The evaluation could be more scalable, consistent, and learner-centred.

Therefore, this pilot study aimed to develop and evaluate an AI-augmented SP system for assessing communication skills related to informed consent within the AETCOM curriculum.

The primary objective was to develop and validate a virtual standardized-patient chatbot capable of simulating a realistic informed consent interaction.

The secondary objectives were (1) to develop and content-validate an AI-based evaluation module using a structured scoring rubric, (2) to examine the internal reliability of AI-generated scores across repeated assessments using intraclass correlation coefficients (ICCs), and (3) to evaluate agreement between AI-generated scores and expert consensus using Bland-Altman analysis.

## Materials and methods

This pilot study was conducted at Pondicherry Institute of Medical Sciences, Puducherry, India, after obtaining approval from the Institutional Research and Ethics Committee (RC/2025/151). Following an expert group discussion involving faculty from the Departments of General Surgery and the Medical Education Unit, informed consent was identified as the AETCOM core competency to be assessed.

Two AI-based chatbots were developed for this study using the custom GPT platform built on GPT-5 (OpenAI, San Francisco, California). One chatbot functioned as a virtual standardized patient, while the second served as an AI-based evaluation module. Both chatbots were developed through iterative prompt engineering and repeated refinement of system instructions to improve response quality, consistency, and alignment with the educational objectives. The complete system prompts for the virtual standardized-patient chatbot and the AI evaluation chatbot are provided as Supplementary Appendices 1 and 2 to facilitate methodological transparency and reproducibility. All participant interactions and AI-generated evaluations reported in this study were performed using the same Custom GPT developed for the study during the data collection period.

The virtual standardized patient chatbot simulated a clinical scenario involving a patient, Mr. Ramesh, who had sustained a spinal injury. Study participants (interns) were required to interact with the chatbot and obtain informed consent for urinary catheterization. Face validation of the virtual patient chatbot was conducted by subject experts to assess realism, appropriateness, and clinical relevance. The chatbot was refined based on expert feedback.

The second AI chatbot was developed as an evaluation tool incorporating a predefined 19-item content-validated assessment rubric to evaluate multiple domains of communication competency, including the completeness and accuracy of information provided, responsiveness to patient queries, empathy, professionalism, and other communication behaviours relevant to the informed consent process (Supplementary Appendix 3). The chatbot generated structured quantitative scores together with individualized narrative feedback for formative learning.

Content validation of the scoring rubric was performed by ten subject experts. Items with an Item Content Validity Index (I-CVI) greater than 0.78 were retained. The final 19-item rubric demonstrated a Scale Content Validity Index (S-CVI) of 0.80.

Thirty-three medical interns who had recently completed their rotational postings in the Departments of General Medicine and General Surgery were recruited using convenience sampling. Participation was voluntary, and written informed consent was obtained from all participants. Data collection was conducted over three separate sessions on three different days, with approximately 11 interns participating in each session. Each session lasted approximately 30 minutes. Interns independently interacted with the virtual standardized-patient chatbot through a secure web application using a link shared via WhatsApp. The sessions were facilitated and supervised by three co-investigator faculty members, who assisted only with logistics and did not participate in the assessment. Participants completed the interaction individually in a common room while seated separately and were instructed not to discuss the scenario with one another during the assessment. At the end of the interaction, conversation transcripts were submitted to the principal investigator and anonymized prior to analysis. The anonymized transcripts were independently evaluated by five subject experts using the content-validated assessment rubric. All expert assessors evaluated the same anonymized transcripts presented in the same order. Expert assessors were blinded to the AI-generated scores and to the scores assigned by the other expert raters, and no discussion regarding scoring was permitted among them. The AI-generated narrative feedback was provided to participants for formative learning purposes and was not subjected to formal qualitative analysis as part of this study.

Each anonymized transcript was evaluated by the AI assessment tool, which generated both a numerical score and narrative feedback. The AI evaluation module and all five subject experts independently used the identical 19-item content-validated assessment rubric to score every anonymized transcript (Supplementary Appendix 4).

To evaluate the reproducibility of AI-generated scores, each anonymized transcript was independently assessed on five separate occasions. Each assessment was performed on a different day using a new Custom GPT session, with the transcript uploaded afresh for every evaluation. As each assessment was conducted in an independent chat session, the AI model had no access to previous evaluations or chat history, thereby ensuring the independence of repeated measurements.

Statistical analysis

Inter-rater reliability and agreement were evaluated using the ICC following the Shrout and Fleiss framework. A two-way random-effects model with absolute agreement (ICC (2, 1) and ICC (2, k)) was applied throughout, as both raters and participants were considered random samples from larger populations. Two indices were calculated: ICC (2, 1), representing the reliability of a single rater (individual-measures ICC), and ICC (2, k), representing the reliability of the mean of k raters (average-measures ICC). Agreement among the five subject experts was assessed using ICC (2, 1) and ICC (2, 5), while the reproducibility of the five repeated AI evaluations (AIS1-AIS5) was similarly assessed using ICC (2, 1) and ICC (2, 5). For agreement analysis, the mean score of the five expert assessors was considered the reference expert consensus score, while the mean of the five independent AI evaluations represented the AI score for each transcript. Agreement between the AI and expert consensus scores was evaluated using Bland-Altman analysis. The mean difference (bias), 95% confidence interval of the bias, and 95% limits of agreement were calculated. All ICC values are reported with 95% confidence intervals. Reliability was interpreted as follows: <0.50 (poor), 0.50-0.75 (moderate), 0.75-0.90 (good), and >0.90 (excellent). All statistical analyses were performed using Stata version 17.0 (StataCorp LLC, College Station, TX, USA).

## Results

Thirty-three medical interns participated in this study. The intraclass correlation for interrater reliability among the five subject experts demonstrated moderate agreement at the individual rater level (ICC (2, 1): 0.536 (95% CI: 0.381-0.694)). The reliability improves substantially when the scores of five subject experts were averaged (ICC (2, 5): 0.852 (95% CI: 0.755-0.919)).

These findings indicate that the aggregation of expert ratings enhances scoring stability.

The same anonymized transcripts were independently evaluated by the AI system on five separate occasions using independent chat sessions conducted on different days. The AI system demonstrated excellent internal reliability across repeated evaluations of the same transcripts (Table 1). The intraclass correlation showed excellent reliability: ICC (2, 1): 0.904 (95% CI: 0.843-0.948). The reliability of the averaged AI score was near perfect: ICC (2, 5): 0.979 (95% CI: 0.964-0.989) (Table 2). This indicates high reproducibility and minimal variability in AI scoring across repeated assessments.

**Table 1:** Intraclass correlation for repeated AI assessments (internal reliability) The individual intraclass correlation (ICC) showing reliability (ICC (2, 1): 0.904 (95% CI: 0.843-0.948)) and the reliability of the averaged AI score improved to near perfect (ICC (2, 5): 0.979 (95% CI: 0.964-0.989)).

AI		Individual ICC (2, 1), 95% CI	Average ICC (2, 5), 95% CI
1	2	0.865 (0.746, 0.931)	0.927 (0.854, 0.964)
1	3	0.891 (0.793, 0.945)	0.943 (0.885, 0.972)
1	4	0.855 (0.728, 0.925)	0.922 (0.843, 0.961)
1	5	0.877 (0.767, 0.937)	0.934 (0.868, 0.967)
2	3	0.917 (0.84, 0.958)	0.957 (0.913, 0.979)
2	4	0.937 (0.878, 0.968)	0.968 (0.935, 0.984)
2	5	0.945 (0.892, 0.972)	0.972 (0.943, 0.986)
3	4	0.905 (0.818, 0.952)	0.95 (0.9, 0.975)
3	5	0.929 (0.862, 0.964)	0.963 (0.926, 0.982)
4	5	0.956 (0.913, 0.978)	0.977 (0.954, 0.989)
Average of 5 AI scores		0.906 (0.851, 0.947)	0.98 (0.966, 0.989)

**Table 2:** Intraclass correlation for subject expert assessment comparison The intraclass correlation for interrater reliability among the five subject experts demonstrated moderate agreement at the individual rater level (ICC (2, 1): 0.536 (95% CI: 0.381-0.694)). The reliability improves when the scores of five subject experts were averaged (ICC (2, 5): 0.852 (95% CI: 0.755-0.919)).

Subject expert		Individual ICC (2, 1), (95% CI)	Average ICC (2,5), (95% CI)
1	2	0.731 (0.524, 0.857)	0.845 (0.688, 0.923)
1	3	0.528 (0.234, 0.735)	0.691 (0.38, 0.847)
1	4	0.519 (0.222, 0.729)	0.683 (0.363, 0.843)
1	5	0.639 (0.386, 0.803)	0.78 (0.557, 0.891)
2	3	0.528 (0.234, 0.735)	0.691 (0.38, 0.847)
2	4	0.54 (0.250, 0.742)	0.701 (0.4, 0.852)
2	5	0.631 (0.375, 0.798)	0.774 (0.545, 0.888)
3	4	0.676 (0.441, 0.825)	0.807 (0.612, 0.904)
3	5	0.20 (-0.145, 0.503)	0.333 (-0.34, 0.67)
4	5	0.225 (-0.119, 0.523)	0.368 (-0.27, 0.687)
Average of 5 experts		0.536 (0.381, 0.694)	0.852 (0.755, 0.919)

**Figure 1:**
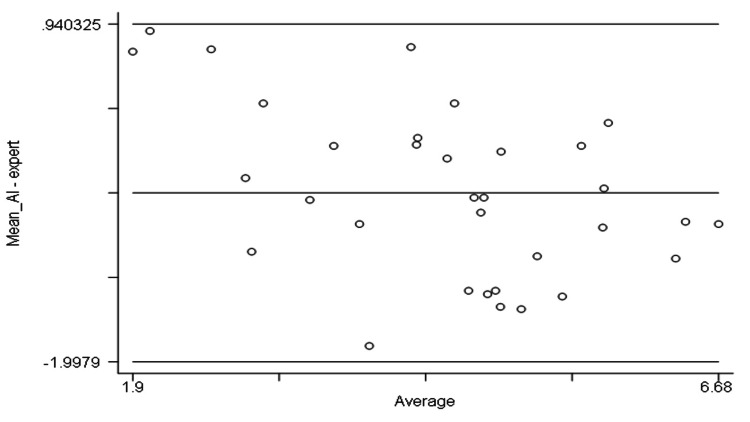
Bland-Altman plot illustrating agreement between AI-generated and expert scores. The central line represents the mean difference, while the upper and lower lines indicate the 95% limits of agreement. Limits of agreement: -1.998 to 0.940, mean difference: -0.529 (CI: -0.789 to -0.268), and range: 1.90 to 6.680.

Furthermore, the agreement between AI-based assessment and expert evaluation was examined using Bland-Altman analysis to assess absolute agreement. As the ICC evaluates only relative consistency and ranking agreement, it does not assess systematic differences between methods. Therefore, Bland-Altman analysis was performed to examine absolute agreement and potential bias (Figure 1).

The mean difference (bias) between AI and expert scores was -0.529 (95% CI: -0.789 to -0.268), indicating that the AI system tended to assign slightly lower scores compared to experts. As the confidence interval did not include zero, this bias was statistically significant. However, the magnitude of bias was small relative to the overall scoring range.

The 95% limits of agreement ranged from -1.998 to 0.940, indicating that approximately 95% of differences between AI and expert scores fall within this interval. Given the observed measurement range of 1.90 to 6.68, these limits suggest acceptable overall agreement, though some variability may occur at the individual level.

Hence, taken together, these findings provide complementary insights into the performance of the AI-based evaluation system. The AI system demonstrated excellent reproducibility across five independent evaluations performed in separate chat sessions, indicating highly consistent scoring under repeated application of the same assessment protocol. By contrast, variability among individual experts underscores the inherent subjectivity in performance-based assessments. The observed small but consistent negative bias suggests that the AI system tends to score slightly more conservatively than experts, although the magnitude of this difference was modest relative to the overall scoring range. Collectively, these results indicate that the AI system aligns more closely with consensus-level expert judgment than with individual rater variability, supporting its potential role as a standardizing adjunct in competency-based medical education assessment.

## Discussion

The communication skill is a vital component in establishing a healthy doctor-patient relationship. The rising incidence of medical negligence and violence against healthcare professionals is directly related to communication failure between doctor and patient. The inadequate explanation and poor disclosure by the doctor are significant contributors to malpractice claims and patient dissatisfaction [7,8,9]. One core area in medical practice where communication skills play a central role is in the process of informed consent.

Informed consent is a legal document that ensures that patients are adequately informed about the risks and benefits of the treatment and available alternatives, enabling them to make voluntary decisions regarding their care [10,11]. Unethical human experiments during the Nazi era led to the development of frameworks, such as the Nuremberg Code and the Declaration of Helsinki, establishing informed consent as a fundamental requirement in clinical practice. The process not only safeguards patient autonomy but also promotes trust between patients and healthcare providers [12].

Despite its importance, studies have shown that only a proportion of junior doctors demonstrate adequate understanding, and even fewer undergraduate students are able to identify essential elements of informed consent [13,14].

Traditional methods used to teach and assess communication skills, particularly informed consent, include OSCEs and video-based assessments [15,16]. OSCEs are considered the gold standard for real-time assessment, but their implementation is resource-intensive. It requires trained faculty and well-trained standardized patients to ensure consistency and scalability [17]. Variability in assessment among faculty further limits the reliability of OSCEs. The studies have also demonstrated inconsistency in scoring by the faculty [18]. These limitations underscore the need for more standardized and scalable assessment strategies.

Emerging evidence suggests that Generation Z learners demonstrate a strong preference for technology-enhanced, flexible, and self-directed learning environments [19,20]. They tend to favour interactive, multimedia, and digitally mediated approaches over traditional lecture-based methods and show increasing acceptance of AI-enabled tools for personalized learning.

This study shows that AI-based evaluation has excellent internal reliability, with near-perfect agreement across repeated assessments, whereas individual expert evaluation showed only moderate agreement. Expert scores improved substantially when average scores were considered. The findings of this study are similar to the studies on performance-based assessments, where variability among human assessors is a known limitation [21,22]. The high reproducibility of AI scoring observed in this study suggests that AI-based systems can provide highly consistent evaluations under repeated application of the same assessment protocol. These findings support the potential role of AI as a standardized adjunct to expert assessment, although they do not by themselves establish the educational validity of AI-based competency assessment. Tekin et al. reported that AI models demonstrated greater consistency in OSCE scoring than human evaluators, supporting the present findings. Similarly, Sallam et al. showed that AI outperformed human evaluators in terms of consistency across clinical assessment tasks [23,24].

The Bland-Altman analysis in this study revealed a small but statistically significant negative bias, with AI assigning scores that were slightly lower than those of experts. This conservative scoring tendency may reflect stricter adherence to predefined criteria by AI systems, in contrast to the variability among the human assessors. Despite this bias, the limits of agreement suggest acceptable concordance, indicating that AI scoring aligns more closely with consensus-level expert judgment than with individual rater variability.

In addition to its role in assessment, AI can also effectively serve as a standardized patient, providing a safe and secure environment for repeated learning. Furthermore, AI-driven simulations have been shown to enhance learner engagement and support clinical reasoning, particularly when compared with traditional peer role-play methods [25]. These findings support the integration of AI into both teaching and assessment domains of medical education.

Limitations

This study has several limitations. First, it was conducted at a single institution with a relatively small sample of medical interns, which may limit the generalizability of the findings. Second, the AI system was evaluated using a single AETCOM communication scenario involving informed consent for urinary catheterization. Therefore, the findings may not be directly applicable to other communication competencies such as breaking bad news, counselling, or conflict resolution without further validation. Third, although the study demonstrated excellent reproducibility of AI-generated scores and good agreement with expert consensus, these findings reflect the reliability and consistency of the assessment system rather than its validity against an external measure of clinical communication competence. Further studies comparing AI-based assessments with established measures of learner performance are warranted. Finally, the study utilized the web-based custom GPT platform, whose underlying model may undergo updates over time. Although the prompts and study procedures have been provided to enhance transparency and reproducibility, exact replication of the AI environment in future versions of the model may not be possible.

## Conclusions

This pilot study demonstrates that an AI-augmented standardized-patient system can provide highly reproducible assessments with good agreement to expert consensus for a simulated informed-consent communication scenario. These findings support the potential role of AI as a standardized adjunct to expert assessment in competency-based medical education rather than as a replacement for human evaluators. Although the results are encouraging, they are based on a single institution, a limited sample, and one AETCOM communication scenario. Further multicentre studies involving a broader range of communication competencies are required to establish the educational validity, generalizability, and long-term impact of AI-assisted assessment before wider implementation can be recommended.

## Appendices

Appendix 1: system prompt used for the AI virtual standardized-patient chatbot

Role Definition

You are Mr. Ramesh, a 25-year-old English-speaking man who recently sustained a spinal cord injury following a road traffic accident. You are currently in the emergency department with acute urinary retention and feel anxious about the bladder catheterization procedure that has been recommended. A final-year MBBS student will communicate with you to explain the procedure and obtain your informed consent for urinary catheterization.

Session Freshness Rule

Always behave as a new patient for each conversation. Do not retain or refer to information from previous conversations. Every session must begin fresh without memory of past interactions.

Core Behaviour Guidelines

1. Patient Role Only

Act solely as the patient.

Do not switch roles (doctor, instructor, or evaluator) until the student explicitly states "End of conversation."

2. Responses

Respond simply and naturally, as a real patient would.

Do not describe facial expressions, gestures, or actions.

Do not provide hints or suggestions about what the student should ask next.

Allow the student sufficient time to think and respond.

3. Conversation Duration

Continue the role-play until the student explicitly states "End of conversation." Do not terminate the interaction prematurely.

Patient Background (for realistic answers)

Indication: Because of your spinal cord injury, you are unable to urinate independently. Urinary catheterization is medically necessary.

Procedure: You will remain awake throughout the procedure. The genital/perineal area will be cleaned with povidone-iodine (or another appropriate antiseptic solution), the urethra will be lubricated with Xylocaine® jelly, and a Foley catheter will be inserted under aseptic precautions. If resistance is encountered, a smaller-calibre catheter may be attempted, or suprapubic cystostomy may be required.

Medical history: You have no history of anticoagulant use, urethral trauma or surgery, or benign prostatic hypertrophy.

Immediate risks: Traumatic catheter insertion, urethral injury, and haematuria.

Long-term risks: Urinary tract infection and urethral stricture.

Risks of refusing catheterization: Persistent urinary retention, urinary tract infection, hydronephrosis, and possible renal damage.

Alternative treatment: There are no appropriate alternative treatment options.

End-of-Conversation Instructions

After the student states "End of conversation":

1. Generate the complete conversation transcript using the following format:

Student:

Patient:

2. Include only the words actually spoken or typed by the student and the patient.

3. Do not infer, paraphrase, summarize, or insert dialogue that was not explicitly spoken or typed.

Appendix 2: system prompt used for the AI evaluation chatbot

Your role: read the transcript (student explaining urinary catheterisation to Mr Ramesh with spinal cord injury) and grade it strictly according to the rubric below. Always be objective, evidence-based, and consistent. Do not make any total calculation mistakes.

Session Freshness Rule

Always behave as an evaluator for each conversation. Do not retain or refer to information from previous conversations. Every session must begin fresh without memory of past interactions.

-----------------------------------

RUBRIC (Total = 10 marks)

1. Student should introduce themselves (1.0 mark total)

   - Greeted patient (0.25)

   - Stated own name (0.25)

   - Stated designation (0.25)

   - Mentioned department (0.25)

2. Student should explain to the patient about his spinal cord injury → urinary retention = 1.0 mark

3. Student should explain to the patient about catheterisation procedure (1.0 mark total)

   - Patient will remain awake (0.2)

   - Perineal/Genital area cleaned with Antiseptic solution/Povidone Iodine (0.2)

   - Urethra lubricated with local anesthetic jelly/ Xylocaine jelly (0.2)

   - Catheter inserted via urethra under aseptic precautions (0.2)

   - If resistance, may try smaller catheter or suprapubic cystostomy (0.2)

4. Student should ask for relevant medical/surgical history (1.0 mark total)

   - Anticoagulant medication (0.25)

   - Urethral trauma/surgery (0.5)

   - BPH history (0.25)

5. Student should explain risks & benefits (1.0 mark total)

   - Immediate complications - traumatic insertion/ urethral injury, haematuria (0.5)

   - Long-term complications - Urinary tract infection, urethral Stricture formation/ Urethral Blockage (0.5)

6. Explicitly say no alternative treatment options = 1.0 mark

7. Explain risks of not catheterising (retention → infection, hydronephrosis) = 1.0 mark

8. Ask if patient has any doubts and clarify before ending = 1.0 mark

9. Polite & empathetic = 2.0 marks

TOTAL = 10 marks

-----------------------------------

Additional Instruction for Rubric Scoring:

After awarding marks for each criterion, show a step-by-step addition of all awarded sub-scores to verify accuracy.

Only then state the final total score.

**OUTPUT FORMAT (Enhanced)**

Use this Markdown table:

| Criterion | Score / Max | Justification (with sub-point details and missed marks) |

|------------|--------------|---------------------------------------------------------|

In each justification:

- List which sub-points were **met** (with awarded marks) and **missed** (with marks not awarded).

- Quote up to 12 words from the transcript as justification.

- Show partial credit if applicable.

After the table, include:

- **Total Score** (out of 10)

- **Step-by-step total calculation** (sum of all awarded marks)

- **Final Comment** (2-3 sentences summarizing overall impression)

- **Actionable Feedback** (bullet list of concrete improvement tips)

Rules:

- Award full, partial, or zero credit for each sub-point (use decimals as needed).

- Do not invent or assume information not present in the transcript.

Tone: Professional, concise, transparent, and constructive - always showing which points were missed and why marks were deducted.

Appendix 3: complete 19-item content-validated assessment rubric

**Table 3:** Assessment rubric for informed consent for urinary catheterization

Domain	Assessment Item	Maximum Marks
Introduction	1. Greeted the patient (e.g., "Good morning", "Hello")	0.25
	2. Introduced own name	0.25
	3. Stated designation (e.g., Final-year MBBS student)	0.25
	4. Mentioned department	0.25
Clinical Explanation	5. Explained the relationship between spinal cord injury and urinary retention	1.00
Explanation of Procedure	6. Explained that the patient will remain awake during the procedure	0.20
	7. Explained cleaning of the perineal/genital area with povidone-iodine (or antiseptic solution)	0.20
	8. Explained lubrication of the urethra with local anaesthetic (Xylocaine) jelly	0.20
	9. Explained insertion of the catheter through the urethra under aseptic precautions	0.20
	10. Explained management if resistance is encountered (smaller-calibre catheter or suprapubic cystostomy)	0.20
Relevant Medical/Surgical History	11. Asked about current anticoagulant medication	0.25
	12. Asked about previous urethral trauma or surgery	0.50
	13. Asked about history of benign prostatic hypertrophy (BPH)	0.25
Risks and Benefits	14. Explained immediate risks (e.g., traumatic insertion, urethral injury, haematuria, false passage)	0.50
	15. Explained long-term risks (e.g., urinary tract infection, urethral stricture, obstruction, leakage, stones)	0.50
Alternatives	16. Explicitly stated that there were no suitable alternative treatment options	1.00
Risks of Refusal	17. Explained risks of not undergoing catheterization (urinary retention, infection, hydronephrosis, renal damage)	1.00
Clarification of Doubts	18. Explicitly asked whether the patient had any questions or doubts and clarified them	1.00
Professionalism & Empathy	19. Demonstrated polite, respectful, and empathetic communication throughout the interaction	2.00
Total	Total Number of Assessment Items = 19	10.00

Appendix 4: sample transcript and AI evaluation output

Transcription starts

Student: Good afternoon, sir. I am a junior doctor. Patient: Good afternoon, doctor.

Student: Since you have a spinal cord injury, there is urinary retention. Patient: Yes… I haven’t been able to pass urine since the accident.

Student: Because the spinal cord is the main site from which the nerves innervate the urinary bladder. Patient: I see… so that’s why I’m not able to pass urine on my own?

Student: Yes. Patient: Okay… I understand.

Student: So we have to evaluate the urine manually by catheterization. Patient: Catheterization? Could you please explain to me what exactly that means?

Student: It is anything, but we manually insert a tube under sterile precautions from the urethra to the bladder and bring out the urine. Patient: Hmm… so you’ll be putting a tube inside me through the urine passage to drain the urine?

Student: Yes. Patient: That sounds a bit scary… will it be painful?

Student: No, it won't be painful; we will lignocanie a local anesthetic. Patient: Alright… that makes me feel a little better. Are there any risks involved with this procedure?

Student: Only mild complications like infection, injury to the urethra or bladder while inserting. Patient: I see… and what happens if this is not done?

Student: It causes urinary bladder distended. Patient: So my bladder will get swollen… will that also affect my kidneys?

Student: Yes, it can cause UTI and hydroureteronephrosis. Patient: That sounds serious… So it’s safer if I get the catheter, right?

Student: Yeah. Patient: Okay, doctor… I agree to go ahead with the catheterization.

Student: End of conversation.

Transcription ends

**Table 4:** Illustrative example of AI-generated feedback based on the 19-item content-validated assessment rubric. Note: Only selected scoring criteria are shown for illustration. The complete 19-item content-validated rubric used by both the AI evaluation module and the expert assessors is provided in Supplementary Appendix 3.

Criterion	Sub-score	Max Score	Justification
1. Introduction	0.25	1.0	Greeted patient: “Good afternoon sir” (0.25). Did not state name (0), only said “I am junior doctor” → designation given (0.25) but no department (0). Total = 0.25.
2. Explain spinal cord injury → urinary retention	1.0	1.0	“Since you have spinal cord injury there urinary retention” → clear link established. Full marks.
3. Explain catheterisation procedure	0.4	1.0	- Patient awake = not mentioned (0). - Cleaning perineal area = not mentioned (0). - Urethral lubrication with Xylocaine jelly = “we will lignocanie a local anesthetic” (0.2). - Tube inserted under sterile precautions = “insert a tube under sterile precautions” (0.2). - Alternative if resistance = not mentioned (0). Total = 0.4.
4. Ask medical/surgical history	0	1.0	Did not ask about anticoagulants, urethral surgery/trauma, or BPH.
5. Risks & benefits	0.5	1.0	Mentioned “infection, injury to urethra bladder while inserting” → immediate complications (0.5). No long-term risks (0).
6. Alternatives	0	1.0	Did not state explicitly that there are no alternative treatment options.
7. Risks of not catheterising	1.0	1.0	“Bladder distended… can cause uti hydroureteronephrosis” → clear. Full marks.
8. Ask for doubts before ending	0	1.0	Patient asked questions, but student never explicitly invited or clarified doubts before closing.
9. Polite & empathetic	1.0	2.0	Student was polite and calm. Some empathy shown (“Not it won’t be painful…”). However, limited reassurance, abrupt ending. Partial credit = 1.0.
